# Anti-N-methyl-D-aspartate receptor encephalitis: characteristics and rapid diagnostic approach in the emergency department

**DOI:** 10.1186/s12883-022-02752-9

**Published:** 2022-06-18

**Authors:** Jin Xu, Na Zhao, Hongzhi Guan, Joseph Harold Walline, Huadong Zhu, Xuezhong Yu

**Affiliations:** 1 Emergency Department, State Key Laboratory of Complex Severe and Rare Disease, Peking Union Medical College Hospital, Chinese Academy of Medical Sciences and Peking Union Medical College, Beijing, China; 2 Department of Anesthesiology, Beijing Obstetrics and Gynecology Hospital, Capital Medical University, Beijing, China; 3 Department of Neurology, Peking Union Medical College Hospital, Chinese Academy of Medical Sciences and Peking Union Medical College, Beijing, China; 4 Centre for the Humanities and Medicine, The University of Hong Kong, Hong Kong, China

**Keywords:** Anti-N-Methyl-D-Aspartate Receptor Encephalitis, Diagnostic Errors, Emergency Department, Oligoclonal Bands, Teratoma

## Abstract

**Background:**

Anti-N-methyl-D-aspartate receptor (anti-NMDAR) encephalitis is a common type of autoimmune encephalitis. Patients with this condition are frequently very ill but are often misdiagnosed in the Emergency Department (ED). The objective of this study was to analyze the clinical characteristics of anti-NMDAR patients in the ED and to identify any associations with a diagnosis of anti-NMDAR encephalitis.

**Methods:**

We performed a retrospective analysis of a prospectively obtained cohort of ED patients from May 2011 to December 2017. We identified patients diagnosed with anti-NMDAR encephalitis in this cohort and extracted key patient characteristics and clinical data, including patient gender, age, presentation, modified Rank Score (m-RS), laboratory test results, significant treatments, and mortality.

**Results:**

Eighty-seven patients with anti-NMDAR encephalitis were identified. 54 (62.1%) were female, 23 (26.4%) were < 18 years old, 14 (16.1%) had teratoma, and 45 (51.7%) had an m-RS ≥ 4. Fever, altered mental status, and seizures were the most common symptoms, with a > 50% incidence of each symptom in the cohort. The sensitivity of CSF oligoclonal band (OB) testing was 78.9%. 22 (25.3%) were admitted to the ICU, 20 (23.0%) patients were intubated, but only one patient died (1.1%). 47 (54.0%) were misdiagnosed prior to ED arrival. All patients underwent immunotherapy as first-line treatment for anti-NMDAR encephalitis.

**Conclusions:**

A majority of anti-NMDAR encephalitis patients presenting to the ED were female and were likely to be misdiagnosed prior to arrival. Patients with symptoms of fever, altered mental status, and seizures need a lumbar puncture, including CSF OB testing, for definitive diagnosis.

## Background

Anti-N-methyl-D-aspartate receptor (anti-NMDAR) encephalitis is a common type of autoimmune encephalitis, where pathogenic autoantibodies are directed against the NR1 subunit of the N-methyl-D-aspartate (NMDA) receptor. NMDA receptors are found in the hippocampus of the human brain, where they are involved in learning and memory [1]. Anti-NMDAR autoantibodies attack these receptors, leading to neuropsychiatric symptoms [2–5]. Anti-NMDAR encephalitis predominantly affects children and young adults and surpasses the frequency of any individual virus encephalitis in young people [6]. Furthermore, 53%-77% of anti-NMDAR encephalitis patients are severely ill and require intensive care unit (ICU) admission [7, 8]. In a retrospective study, anti-NMDAR encephalitis accounted for 1% of all admissions for young adults to ICUs [9]. However, prompt diagnosis and treatment lead to improvement or full recovery in most cases [10]. As some of these patients (especially those who are severely ill) present to Emergency Departments (EDs), many can be misdiagnosed as having purely psychiatric illnesses on first contact with ED physicians [11]. There may be room to improve on the ED diagnosis for this disease.

As emergency doctors should make differential diagnosis before setting up a primary diagnosis of patients promptly, they must pay attention to many clinical manifestations. Some known related risk factors may help us in differential diagnosis. Except those typical neuropsychiatric manifestation of anti-NMDAR encephalitis, gender and teratoma are the first concern of patients with anti-NDMAR encephalitis, as this disease was first reported in four young women with ovarian teratoma in 2005 [12], in whom anti-NMDAR antibody were first detected. After that, another eight female patients with the similar neurological symptoms were found with anti-NMDAR antibody and seven of whom also had ovarian teratoma [13]. Second, about 70% of patients with anti-NMDAR encephalitis have prodromal symptoms consisting of fever, headache, nausea, vomiting, diarrhea, et al [14]. However, we did not know details of these prodromal symptoms like how many patients experienced fever, how long it lasted. And these may be regarded as clue of diagnosis for emergency doctors at first medical contact. Infectious encephalitis is a very important one need to be differentiated by emergency doctors, especially the herpes simplex encephalitis which regarded as one trigger of autoimmune encephalitis [15]. But it’s very hard to differentiate virus encephalitis and autoimmune encephalitis as their clinical manifestation and routine ancillary test sometimes overlap unless by using CSF-PCR and CSF autoimmune antibodies.

So, in this study, we sought to verify these risk factors and find more clues to the diagnostic approach of anti-NMDAR encephalitis in ED. We conducted a cohort study, retrospectively analyzed patients diagnosed with anti-NMDAR encephalitis, who experienced ED visits in our hospital, and summarized their clinical manifestations, imaging, and laboratory findings to explore those risk factors which help differential diagnosis in ED.

## Methods

Our institution is a national referral center for complicated diseases, and many encephalitis cases are seen in our ED. Since we have seen many anti-NMDAR encephalitis patients in our ED, we sought to establish a prospective anti-NMDAR encephalitis cohort to learn more about this patient population.

Three hundred twenty-one patients were enrolled in a prospective anti-NMDAR encephalitis cohort between May 2011 and December 2017. We retrospectively analyzed all patients who were admitted through the ED in this cohort. Patient characteristics, including gender, age, presenting complaint(s), modified Rank Score (m-RS), ancillary test results, treatments, and follow up results were recorded for analysis. The m-RS is widely used in the neurology literature to measure functional independence as part of a severity assessment. We recorded m-RS in patients admitted to the ED resuscitation room for ECG/BP/SpO2 monitoring after initial clinical assessment by an ER physician.

Additional data collected included: (1) symptoms upon ED presentation, including: abnormal (psychiatric) behaviors or cognitive dysfunction, speech dysfunction (pressured speech, paucity of speech, or mutism), seizures, movement disorders (including dyskinesia or abnormal posturing), decreased level of consciousness, autonomic dysfunction or central hypoventilation, fevers, or headaches; (2) m-RS; (3) ancillary tests, including: complete blood count (CBC), cerebrospinal fluid (CSF) results, anti-NMDAR serum antibody level, electroencephalogram (EEG), and magnetic resonance imaging (MRI); (4) time and date of anti-NMDAR diagnosis, including any possible misdiagnoses and relation to symptom onset; (5) patient monitoring; (6) ICU admission or airway intubation; (7) treatments, including corticosteroids or intravenous immunoglobulin (IVIG); (8) presence of a teratoma and timing of removal surgery; (9) survival and follow-up results.

Continuous variables were presented as medians with quartiles, and categorical variables as frequencies with percentages. Median m-RS’s were compared using the Mann–Whitney rank sum test.

## Results

After review of the database, 87 ED patients with anti-NMDAR encephalitis were included in the analysis. Of these 87 patients, 54 (62.1%) were female, and 23 (26.4%) were < 18 years old (including three patients < 12). Fever and abnormal behavior were the two most frequent symptoms, while a decreased level of consciousness and seizures were the next two most frequent symptoms (see Table 1).

**Table 1:** Anti-NMDAR encephalitis ED patient characteristics

Patient Characteristics	N (%)
**Gender**	
Female	54 (62.1%)
Male	33 (37.9%)
**Age**	
Adult = ≥ 18 y/o	64 (73.6%)
Pediatric < 18 y/o	23 (26.4%)
**Fever**	58 (66.7%)
**Abnormal behavior**	53 (60.9%)
**Decreased consciousness**	49 (56.3%)
**Seizures**	44 (50.6%)
**Headache**	33 (37.9%)
**Speech Dysfunction**	19 (21.8%)
**Movement disorder**	18 (20.7%)
**Autonomic dysfunction**	6 (6.9%)

**Fig. 1:**
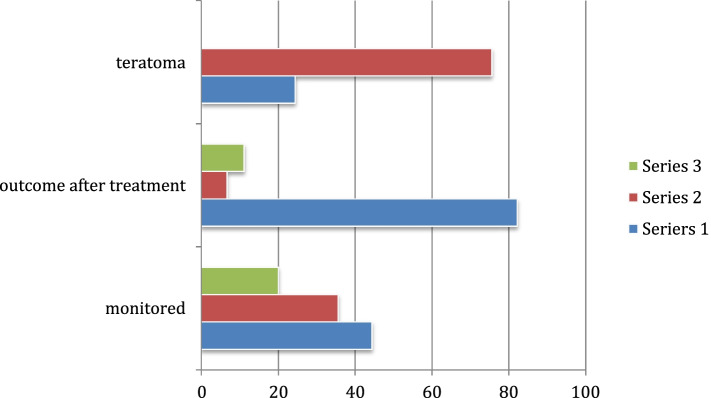
In patients with m-RS ≥ 4, 24.4% patients with teratoma, most of them needed monitoring, but with good outcome. * Teratoma: 11 patients positive (blue) and 34 patients negative (red). Outcome after treatment: 37 patients went into remission (blue), 3 patients had no response (red), and 5 patients’ data were lost (green). Monitored: 20 patients were monitored and intubated (blue), 16 patients were monitored only (red), and 9 patients had no monitoring (green)

84 (96.6%) patients had complete m-RS records, and 45 (51.7%) had an m-RS ≥ 4, of which 36 patients were admitted into the ED resuscitation area and received Electrocardiography (ECG), Saturation of peripheral Oxygen (SpO2), and Blood Pressure (BP) monitoring. Of these 36 monitored patients, 20 were subsequently intubated, placed on mechanical ventilation, and admitted to an ICU. Out of 87 total patients, only one patient died (1.1%). Of the 45 patients with an m-RS ≥ 4, 11 patients had ovarian teratomas, with one case of relapse and no deaths. 37 patients had low m-RS scores (0–3), of which five patients were lost to follow-up, but no deaths were recorded otherwise (see Fig. 1).

**Fig. 2:**
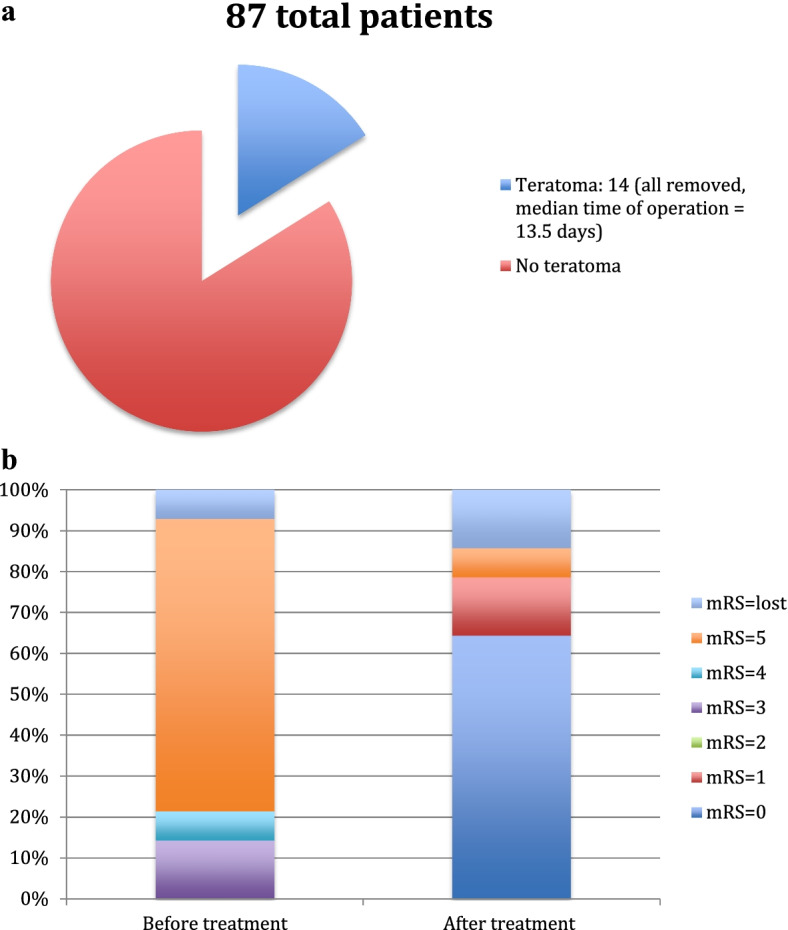
Patients with teratomas, 78.6% had m-RS >  = 4, almost all of them went into remission except one after operation and first line treatment

Overall, 14 (16.1%) patients had teratomas, all of which had teratoma removal operations, with the earliest operation being carried out five days following the diagnosis of anti-NMDAR encephalitis, while the latest operation was 42 days after diagnosis (median 13.5 days) (see Fig. 2a). Of these 14 patients, 10 patients had an m-RS score of 5, with others having lower scores (see Fig. 2b). In the group of patients without a teratoma, the median m-RS score was 3 (3, 5), *P* = 0.002. After surgery, almost all anti-NMDAR patients with a teratoma were in remission (except for one, who had a presenting m-RS = 5) (see Fig. 2b).

44 (50.6%) patients were admitted to the ED resuscitation area and received ECG, SpO2, and BP monitoring after being assessed by an ED physician. Of these 44 patients, 21 presented to the ED due to epileptic seizures, five due to autonomic dysfunction or hypoventilation, five for a decreased level of consciousness, four after teratoma removal operations, and 10 patients did not have any reason listed on their emergency medical records. Of the 44 monitored patients, 20 patients were intubated, 22 were admitted to the ICU, and one patient died (see Table 2).

**Table 2:** Reasons for starting ED monitoring

Reason for monitoring	N (%)
**Patients were monitored**	44 (50.6%)
Due to epileptic seizure	21 (24.1%)
^a^Due to autonomic dysfunction or hypoventilation	5 (5.7%)
After operation	4 (4.6%)
Due to decreased level of consciousness	5 (5.7%)
Unknown reasons	10 (11.5%)
**Patients were not monitored**	43 (49.4%)

^a^1 patient had a contemporaneous epileptic seizure

In reviewing the diagnostic pathway of the 87 total patients, 47 (54.0%) patients had been misdiagnosed before being transferred to our ED. 21 patients were diagnosed as having a viral encephalitis, 17 were diagnosed with a psychiatric condition, including schizophrenia, depression, or anxiety, and 9 had another (not anti-NMDAR encephalitis) diagnosis. 34 patients were diagnosed with anti-NMDAR encephalitis in our ED, with a mean time of diagnosis of 20.5 days. In our study, the sensitivity of an abnormal EEG was 71.2% and CSF oligoclonal band testing (OB) was 78.9%. Notably, finding serum antibodies against the NMDA receptor had a sensitivity of only 66.3%. Additional test results are shown in Table 3.

**Table 3:** Ancillary test results for anti-NMDAR encephalitis ED patients

Ancillary test	Positive (n)	Negative (n)	Data unavailable (n)
**Lymp% in routine blood tests**	7	78	2
**ESR**	22	44	21
**CRP**	24	39	24
**CSF**			
Cell count	52	33	2
WBC	37	48	2
Pro	23	64	0
Glu	11	72	4
Cyto. Exam	43	36	8
OB	45	12	30
**CSF pressure**	24	44	19
**MRI**	34	36	17
**EEG**	47	19	21
**Serum anti-NMDAR antibody**	55	28	4

WBC White blood cell, *Pro*. Protein, *Glu* Glucose

## Discussion

The diagnosis of anti-NMDAR encephalitis is very difficult to make at the point of first medical contact in the ED. To confirm the diagnosis, a positive anti-NMDAR test result from a patient’s CSF is needed, however this may not be available in many medical institutions. Fortunately, anti-NMDAR encephalitis presents with a constellation of characteristic symptoms. In this study, we found fever, abnormal (psychiatric) behaviors, a decreased level of consciousness, and seizures were the most common symptoms, all with an incidence above 50%. Unfortunately, the psychiatric symptoms associated with anti-NMDAR encephalitis can be so significant that many family or emergency physicians refer these patients to psychiatrists for consultation [11, 16]. A total of 87 patients were included in our study, 47 (54%) of whom had misdiagnosed experiences in other hospitals. Unlike in cases of ‘pure’ psychosis, a key hallmark of anti-NMDAR encephalitis is that it is often associated with other symptoms such as fever, decreased level of consciousness, or seizures.

Fever is a common non-specific symptom as mentioned in the Background Part, many articles and authoritative international guidelines take fever as prodromal symptom of anti-NMDAR encephalitis, however, when retrospectively analyzed the emergency medical records of those anti-NMDAR patients, we found up to 58 patients in all (66.7%) had fever as chief complaint. And mostly, fever represent inflammation, it’s one of useful symptoms for emergency doctors to differentiate it from many other diseases like pure psychiatric disease, drug overdose, drug abuse, and hepatic encephalopathy, et al. So, we included the fever as an important symptom in emergency diagnostic procedure in this study.

Therefore, an important first step in the ED is to search for causes of fever, identify possible drug overdoses or other metabolic factors which may lead to decreased levels of consciousness and seizures, but anti-NMDA receptor encephalitis should be on the differential diagnosis for patients with a fever and psychiatric symptoms. Since many patients presenting to the ED with such symptoms will require a lumbar puncture for CSF testing as part of routine ED evaluation, we recommend considering testing for anti-NMDAR antibodies if a clinician suspects the diagnosis and if the medical institution can perform the necessary tests. Meanwhile, more ancillary tests are needed to support the diagnosis of anti-NMDAR encephalitis if antibody testing is unavailable.

In our study, we found the sensitivity of CSF oligoclonal band testing (OB) was 78.9%, and an abnormal EEG 71.2%, but serum antibody against NMDAR had a sensitivity of only 66.3%. Compared with EEG and serum antibody against NMDAR, CSF OB testing is more sensitive and more widely available in many EDs. Graus et al. reported CSF OB sensitivity to be > 60% and could be a useful ancillary test for the clinical diagnosis of anti-NMDAR encephalitis [17]. Also, CSF-OB represents inflammation and synthesis of antibodies in CSF. It is a biomarker to suggest organic disorder diagnosis for emergency physicians. Although OB could be positive in many diseases like multiple sclerosis, acute disseminated encephalomyelitis, and stiff-person syndrome, et al. anti-NMDAR encephalitis has specific symptoms very different from these diseases fortunately. So, we included CSF OB as an important ancillary test and emergency diagnostic procedure.

Patients who presented to the ED were quite ill. According to the m-RS scores, more than half were ≥ 4, and, of these, > 80% received monitoring. Overall, in our study, more than half the patients needed to be monitored after being assessed by an ED physician, and half of those monitored patients were subsequently intubated and admitted to an ICU. We analyzed the reasons why patients were monitored, and we found that seizures, autonomic dysfunction / central hypoventilation, or a decreased level of consciousness were the top three reasons. As is common for many other serious ED conditions, airway and ventilation abnormalities or risks were of most concern in these patients. On a more positive note, only one out of 87 patients with anti-NMDAR encephalitis died.

Female patients suspected of anti-NMDAR encephalitis need tumor screening and teratoma removal operations. Most anti-NMDAR encephalitis patients in our study were female, and 14 had teratomas. In these 14 patients, 10 had an m-RS of 5, and one had a m-RS of 4, so 78.6% had an m-RS score ≥ 4. Patients with teratomas in our study had higher m-RS scores compared to those without teratomas (*p* = 0.002). Given this association, we reviewed the source database (ED and non-ED patients) and found that, out of 201 female patients, 39 had a teratoma (19.4%). Of those patients with a teratoma, 27 had an m-RS of 5, and patients with a m-RS ≥ 4 take up 74.4% of all patients, like the results in this ED study. We strongly suggest that female patients diagnosed with anti-NMDAR encephalitis should receive tumor screening, especially for ovarian tumors such as teratomas. The most common method for this would be via abdominal or pelvic ultrasound, with computer tomography or MRI of the pelvis as possible options as well.

Immunotherapy is the first line treatment for the anti-NMDAR encephalitis: every patient received a combination of steroids and IVIG to treat anti-NMDAR encephalitis. 39 patients received steroid pulse therapy (methylprednisolone 500 ~ 1000 mg daily). All steroid pulse therapy plans were decided after consultation with a neurologist. 84 (96.6%) patients received IVIG, which was sometimes ordered by ED physicians before consultation a neurologist.

According to these findings above, combined with our own routine clinical practice process, we try to draw out a draft of “Rapid Diagnostic Procedure, Treatment Options in ED and Follow-up referrals”. First, patients with typical symptoms [ especially abnormal (psychiatric) behaviors or cognitive dysfunction, decreased level of consciousness, seizures] and prodromal symptoms [especially fevers or headaches] should get physical examination including vital signs and assessment of airway safety. Second, track the medical history to exclude poisoning, psychiatric disease and drug overdose et al. Third, Routine ancillary test including Complete Blood Count (CBC), liver function, kidney function, electrolyte, blood ammonia, arterial blood gas, chest X-ray, and head CT to exclude common infectious disease, electrolytes disturbance, hepatic and pulmonary encephalopathy, other metabolic factors like alcohol poisoning and organic factors like intracranial tumor, cerebral hemorrhage et al. Forth, Acupuncture for CSF test, including CSF-OB. Fifth, if all clinical manifestation and ancillary test above support the probability of encephalitis except infectious ones, send CSF for anti-NMDAR antibody test, make assessment of airway safety and m-RS, if m-RS >  = 4 and airway is not safe, take IVIG and acyclovir as initial treatment, and refer to neurologist for admission to neurological ward or ICU. MRI and EEG are not available in ED of China and most patients in ED are hardly cooperate with these two examinations, therefore, not recommend in emergency diagnostic procedure.

## Limitations

This study had some limitations. First, anti-NMDAR encephalitis is just one of the autoimmune encephalitis types, which also includes α-amino-3-hydroxy-5-methyl-4-isoxazolepropionic acid receptor (AMPAR), γ-aminobutyric acid (GABA), and leucine-rich, glioma-inactivated 1 (LGI1) et.al, among others. Like these other types of autoimmune encephalitis, they all may have some overlap in symptoms and clinical characteristics. As anti-NMDAR was the most common type of autoimmune encephalitis we encountered in our institution, we limited this study to this sub-type. Second, although we suggest patients with common symptoms need a lumbar puncture for CSF testing, including OB testing, this suggestion needs further study to prove its effectiveness, because the specificity of CSF-OB could not be calculated in this observational cohort study. Third, we used the m-RS score as a proxy to assess for clinical severity in this study, but the decision to monitor depends on each patient’s airway and ventilation condition. We believe future prospective studies will help clarify these limitations.

## Conclusions

Although anti-NMDAR encephalitis is hard to diagnose upon first medical contact in the ED, three common symptoms (fever, altered mental status, and seizures) should prompt emergency medicine physicians to perform a lumbar puncture. CSF testing should include testing for OB and anti-NMDAR antibodies. Like other encephalitis patients in the ED, we should pay attention to their mental status, airway protection and ventilation capabilities. As for female patients with diagnosed or suspected anti-NMDAR encephalitis, tumor screening (most often with ultrasound) is recommended. For patients of both genders, neurologist consultant and immunotherapy are the next steps, which can begin in the ED.

## Data Availability

Anonymized data from this study is available from the corresponding author upon reasonable request from any qualified investigator.

## Abbreviations

anti-NMDAR encephalitis: Anti-N-methyl-D-aspartate receptor encephalitis
ED: Emergency Department
m-RS: Modified Rank Score
ICU: Intensive care unit
CBC: Complete blood count
CSF: Cerebrospinal fluid
OB: Oligoclonal band testing
EEG: Electroencephalogram
MRI: Magnetic resonance imaging
IVIG: Intravenous immunoglobulin
ECG: Electrocardiography
SpO2: Saturation of peripheral oxygen
BP: Blood pressure
